# Changes in macroautophagy, chaperone-mediated autophagy, and mitochondrial metabolism in murine skeletal and cardiac muscle during aging

**DOI:** 10.18632/aging.101181

**Published:** 2017-02-26

**Authors:** Jin Zhou, Shu Yun Chong, Andrea Lim, Brijesh K. Singh, Rohit A. Sinha, Adam B. Salmon, Paul M. Yen

**Affiliations:** ^1^ Program of Cardiovascular and Metabolic Disorders, Duke-NUS Medical School Singapore, Singapore 169857; ^2^ Department of Biomedical Science, Nanyang Technological University, Singapore 637551; ^3^ Department of Molecular Medicine, Barshop Institute for Longevity and Aging Studies, University of Texas Health Science Center at San Antonio, San Antonio, TX 78245, USA; ^4^ Geriatric Research, Education and Clinical Center, South Texas Veterans Health Care System, San Antonio, TX 78229, USA; ^5^ Duke Molecular Physiology Institute, Departments of Medicine and Pharmacology and Cancer Biology, Duke University Medical Center, Durham, NC 27710, USA

**Keywords:** aging, muscle, heart, autophagy, chaperone-mediated autophagy (CMA), fatty acid oxidation, ceramide

## Abstract

Aging causes a general decline in cellular metabolic activity, and function in different tissues and whole body homeostasis. However, the understanding about the metabolomic and autophagy changes in skeletal muscle and heart during aging is still limited. We thus examined markers for macroautophagy, chaperone-mediated autophagy (CMA), mitochondrial quality control, as well as cellular metabolites in skeletal and cardiac muscle from young (5 months old) and aged (27 months old) mice. We found decreased autophagic degradation of p62 and increased ubiquitinated proteins in both tissues from aged mice, suggesting a decline in macroautophagy during aging. In skeletal muscle from aged mice, there also was a decline in LC3B-I conjugation to phosphatidylethanolamine (PE) possibly due to decreased protein levels of ATG3 and ATG12-ATG5. The CMA markers, LAMP-2A and Hsc70, and mitochondrial turnover markers, Drp1, PINK1 and PGC1α also were decreased. Metabolomics analysis showed impaired β-oxidation in heart of aged mice, whereas increased branched-chain amino acids (BCAAs) and ceramide levels were found in skeletal muscle of aged mice that in turn, may contribute to insulin resistance in muscle. Taken together, our studies showed similar declines in macroautophagy but distinct effects on CMA, mitochondrial turnover, and metabolic dysfunction in muscle *vs*. heart during aging.

## INTRODUCTION

Sarcopenia and decreased cardiac function are common features of the decline in physical performance associated with aging. Sarcopenia refers to a loss of skeletal muscle mass that is accompanied by a decrease in muscle strength and increased fatigue. Aging in the heart is associated with pathological hypertrophy and thickening of the ventricle wall, leading to decreased cardiac output. Changes in both metabolism and macroautophagy, a lysosomal-dependent degradation process, have been associated with aging in both these tissues [1-6]. These, in turn may contribute to the decline in function observed in these tissues during aging.

Macroautophagy, referred as autophagy in the remainder of this manuscript, is the most studied form of autophagic process. It involves the formation of double-membrane vesicles termed “autophagosomes”, the sequestration of cytosolic substrate within autophagosomes, and the subsequent fusion of autophagosome and lysosome to form autophagolysosomes, where the engulfed macromolecules such as lipid droplets [7, 8], and glycogen [9] are degraded to provide substrates for cellular metabolism as well as damaged proteins and organelles to maintain cellular homeostasis. There is a tight connection between autophagy and life-span, as genetic manipulation to increase expression of specific autophagy genes in lower organisms promotes longevity [10]. Autophagy-deficient mice also mimic several characteristics of age-related diseases [11], suggesting that autophagy is a key mechanism for protecting against cellular damage during aging. Furthermore, autophagy is a critical process for maintaining muscle and heart function, and tissue-specific impairment of autophagy in muscle and heart leads to sarcopenia and cardiomyopathy, respectively [3, 12]. Although there is evidence suggesting a potential role of autophagy in aging, the specific changes in basal levels of autophagy in skeletal and cardiac muscle during the natural aging process and the underlying mechanism(s) have not been well characterized.

Chaperone-mediated autophagy (CMA) is another type of autophagic process that specifically targets proteins that have a KFERQ amino acid recognition sequence for degradation. During CMA, target proteins are first recognized and bound by Hsc70. The resulting complex then is targeted to the lysosomes by binding to the lysosomal CMA receptor LAMP-2A, whereupon the target protein is unfolded and translocated into the lysosomal lumen for degradation [13]. A decline of CMA during aging has been reported to occur in both the liver and central nervous system that is associated with decreased function [14-16]; however, little is known about CMA in other tissues during aging.

Mitochondria are the key organelles for oxidative phosphorylation and ATP production. Mitochondria preferentially use fatty acid or glucose as energy sources depending on the availability of the type of fuel. The fuel selectivity by mitochondria also is subjected to hormonal regulation. During aging, cellular and metabolic stresses can increase generation of reactive oxygen species (ROS) that can damage mitochondria and lead to mitochondrial dysfunction [17, 18], as well as perturb fuel utilization. Thus, mitochondrial quality control is critical for maintaining normal mitochondrial function and metabolism during aging. This quality control can be achieved by mitochondrial fusion, fission, and/or mitochondrial turnover through removal of damaged mitochondria by autophagy (mitophagy) and concomitant biosynthesis of new mitochondria. Currently, little is known about the changes in mitochondrial quality control and relevant metabolites that occur in aged muscle and heart.

In this study, we systematically examined and compared the markers for autophagy, CMA, and mitochondrial quality control in skeletal and cardiac muscle isolated from young and aged mice, as well as performed metabolomics profiling of acylcarnitines, amino acid and ceramides. Our studies confirm that autophagy declines in both the muscle and heart during aging. However, we also show that aging promotes distinct effects on CMA, mitochondrial turnover, and metabolic dysfunction in muscle *vs*. heart.

## RESULTS

### Accumulation of p62 and ubiquitinated proteins in muscle and heart of aged mice

**Figure 1:**
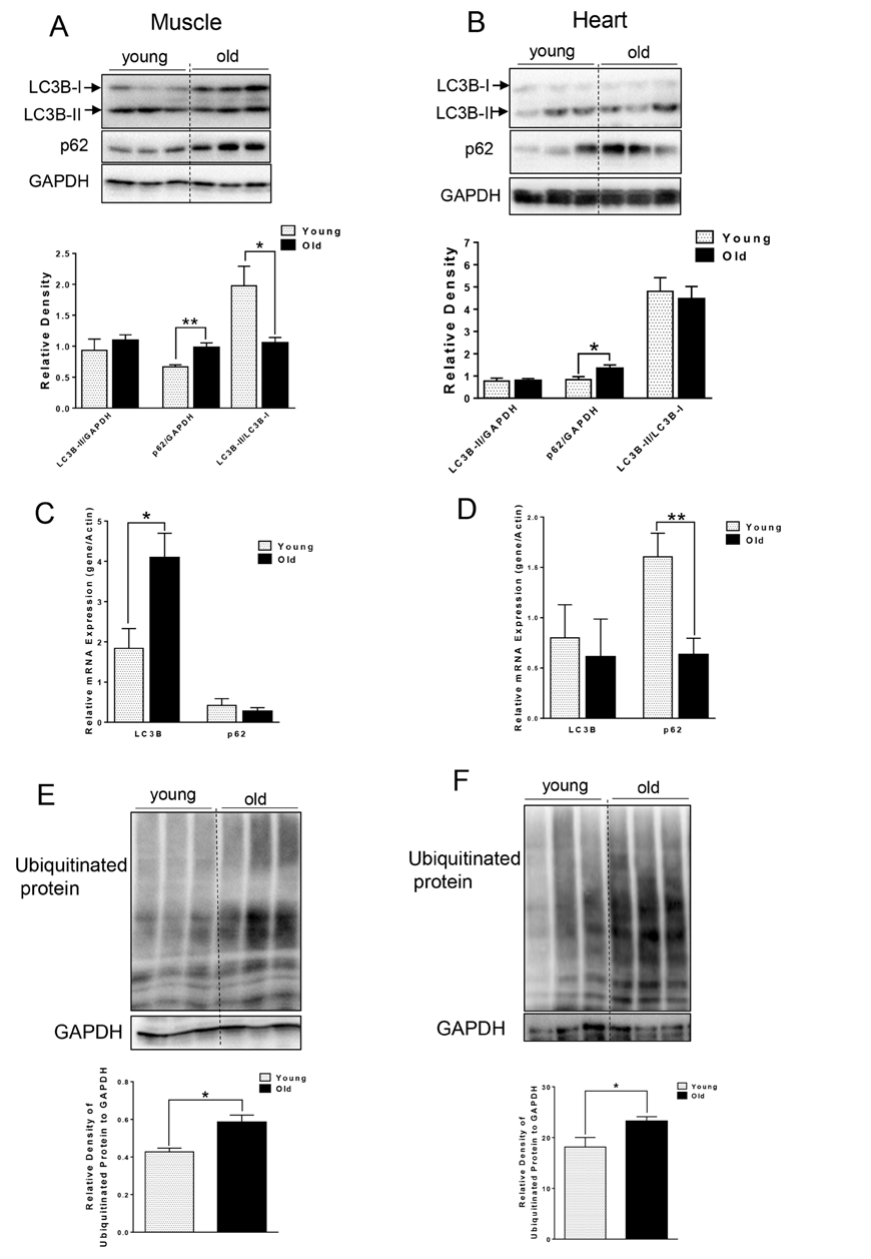
Accumulation of p62 and ubiquitinated proteins in old skeletal and cardiac muscle. (A-B) Immunoblot and densitometric analysis of LC3B and p62 in young and old skeletal muscle (A) and heart (B). (**C-D**) mRNA expression of LC3B, p62 in young and old skeletal muscle (C) and heart (D). (**E-F**) Immunoblot and densitometric analysis of ubiquitinated proteins in young and old skeletal muscle (E) and heart (F). Values are means±SEM for 6 young and 5 old mice in each group. *P < 0.05, **P < 0.01.

We first compared autophagy marker LC3B-II and p62 from muscle and heart tissue that was harvested from young (∼5 month) and aged (∼ 27 month) C57BL/6J mice. Interestingly, we did not observe any difference in the absolute levels of LC3B-II levels *per se*. However, in muscle from aged mice, we observed increased LC3B-I (Fig. 1A), leading to significantly decreased LC3B-II/LC3B-I ratio (Fig. 1A), suggesting an impairment of conjugation of LC3B-I to phosphatidylethanolamine (PE). LC3B mRNA level also was significantly increased in old muscle (Fig. 1C). We also saw a significant increase in p62 protein level in muscle and heart from aged mice (Fig. 1A and B), although the mRNA levels of p62 were unchanged or decreased in muscle or heart tissue from aged mice, respectively. Thus, the increased p62 protein was most likely due to decreased autophagic degradation rather than up-regulation at transcription level (Fig. 1C and D). In support of this notion, there was accumulation of ubiquitinated protein observed in both muscle and heart from aged mice (Fig. 1E and F).

**Figure 2:**
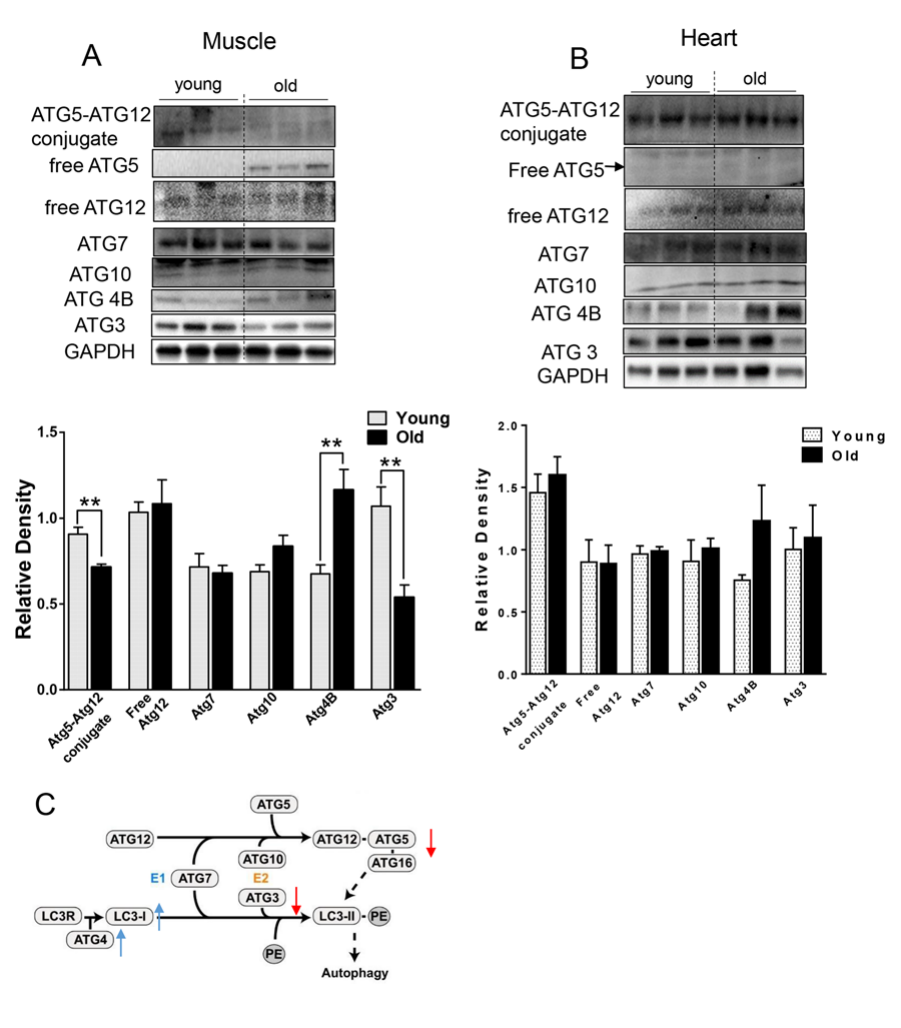
The expression level of ATGs in young and old skeletal and cardiac muscle (**A-B**) Immunoblot and densitometric analysis of ATG proteins in young and old skeletal muscle (A) and heart (B). (**C**) A diagram summarized the change of ATG proteins involved in the two ubiquitin-like conjugation systems in old muscle. Adapted under the terms of the Creative Commons Attribution License from [45], published by the BMC group. Copyright remains with the original authors. Values are means±SEM for 6 young and 5 old mice in each group. *P < 0.05. **P < 0.01.

We next checked the levels of relevant ATG proteins from the two ubiquitin-like systems that are involved in the conjugation of LC3B-I to PE [19]. ATG7 and ATG10 protein levels were unchanged, but ATG4B protein was increased (Fig. 2) in the muscle from aged mice. Additionally, we observed decreased ATG5-ATG12 conjugation (Fig. 2A). Since free ATG5 protein level was increased and ATG12 was unchanged (Fig. 2A), the decrease in ATG5-ATG12 conjugate was due to defective conjugation, rather than decreased ATG5 or ATG12 protein levels. Protein levels of ATG3, the E2-like enzyme, also were decreased (Fig. 2A). Of note, these specific changes were not observed in heart tissue from aged mice (Fig. 2B).

### Decreased protein level of LAMP-2A and Hsc70 in muscle but not heart in aged mice

**Figure 3:**
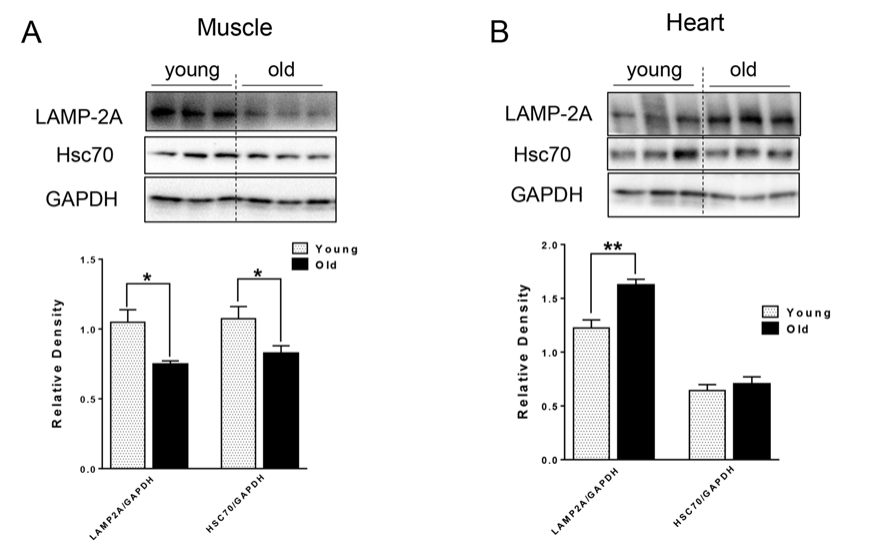
The protein level of CMA markers in young and old skeletal and cardiac muscle. (A-B) Immunoblot and densitometric analysis of LAMP-2A and Hsc70 proteins in young and old skeletal muscle (A) and heart (B). Values are means±SEM for 6 young and 5 old mice in each group. *P < 0.05. **P < 0.01.

For CMA, the substrate proteins are recognized by chaperone Hsc70 and co-chaperones, which then target the resulting complex for lysosomal degradation by binding to lysosomal receptor LAMP-2A [13] located on the lysosomal membrane. We next examined the

levels of CMA markers, and found different effects of aging on CMA makers in the muscle and heart tissue of aged mice. In particular, there were decreased LAMP-2A and Hsc70 protein levels in the muscle of aged mice (Fig. 3A), that could lead to decreased CMA. In contrast, LAMP-2A protein level was increased and Hsc70 protein was unchanged in heart tissue from aged mice (Fig. 3B).

### Decreased markers for mitochondrial quality control in muscle but not heart in aged mice

**Figure 4:**
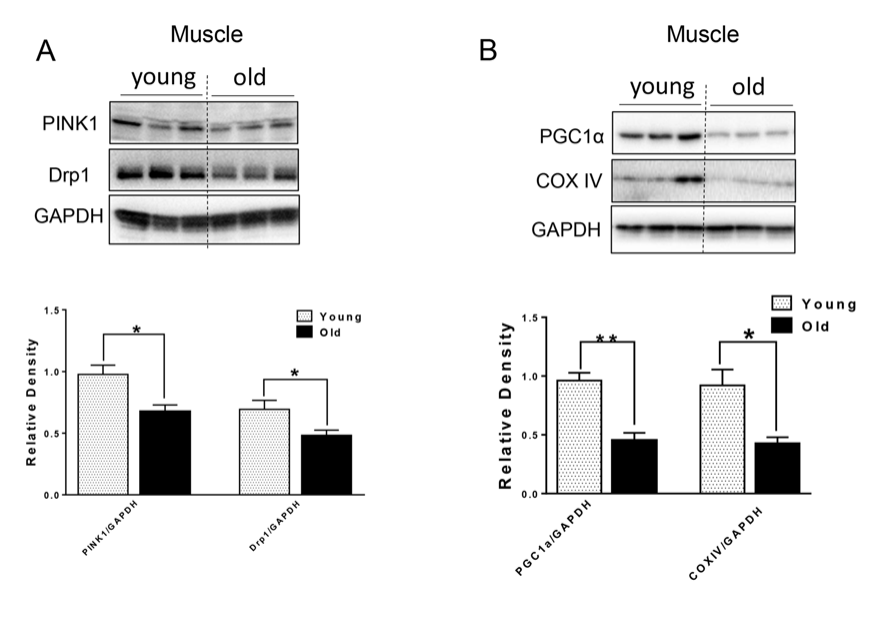
The protein level of mitochondrial quality control markers in young and old skeletal muscle (**A**) Immunoblot and densitometric analysis of PINK1 and Drp1. (**B**) Immunoblot and densitometric analysis of PGC1 α and COXIV. Values are means±SEM for 6 young and 5 old mice in each group. *P < 0.05. **P < 0.01.

Defective autophagy caused by genetic interference leads to not only the accumulation of p62 and ubiquitinated proteins, but also mitochondrial dysfunction in both the muscle and heart [3, 12, 20]. We next examined markers for mitochondrial quality control. In muscle from aged mice, Drp1, a protein associated with mitochondrial fission and subsequent mitophagy [21]; and PINK1, a protein that identifies damaged mitochondrial and targets its degradation specifically by mitophagy [22], were both decreased relative to young murine muscle (Fig. 4A). PGC1α, a key factor for mitochondrial biogenesis [23], and mitochondrial protein COX IV also were decreased in muscle from aged mice (Fig. 4B), indicating that there was decreased mitochondrial biogenesis in muscle from aged mice. The levels of these proteins were unchanged in heart tissue from aged mice (Supplementary Fig. S1).

### Decreased acylcarnitines in the heart but increased BCAA and ceramides in the muscle from aged mice

**Figure 5:**
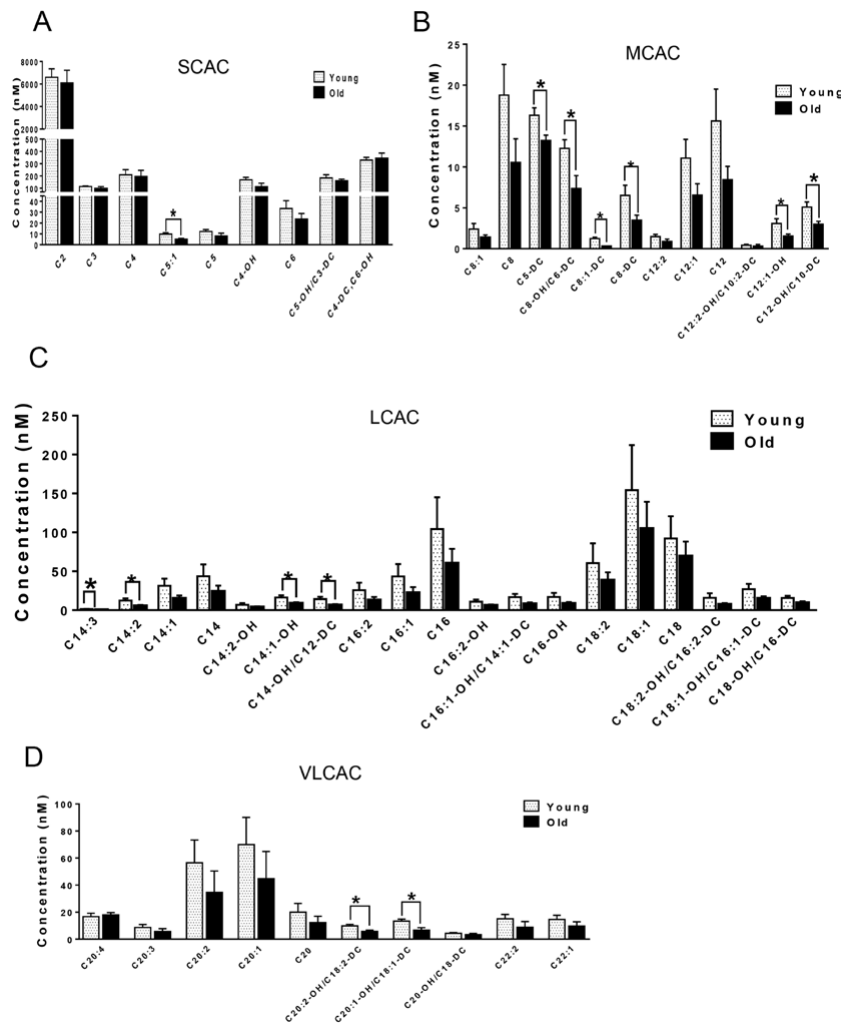
Metabolomic analysis of cardiac acylcarnitines from young and old mice. (A-D) Metabolomic profiles of SCACs, MCACs, LCACs, VLCACs levels in cardiac muscle from young and old mice. Values are means±SEM for 6 young and 5 old mice in each group. *P < 0.05.

**Figure 6:**
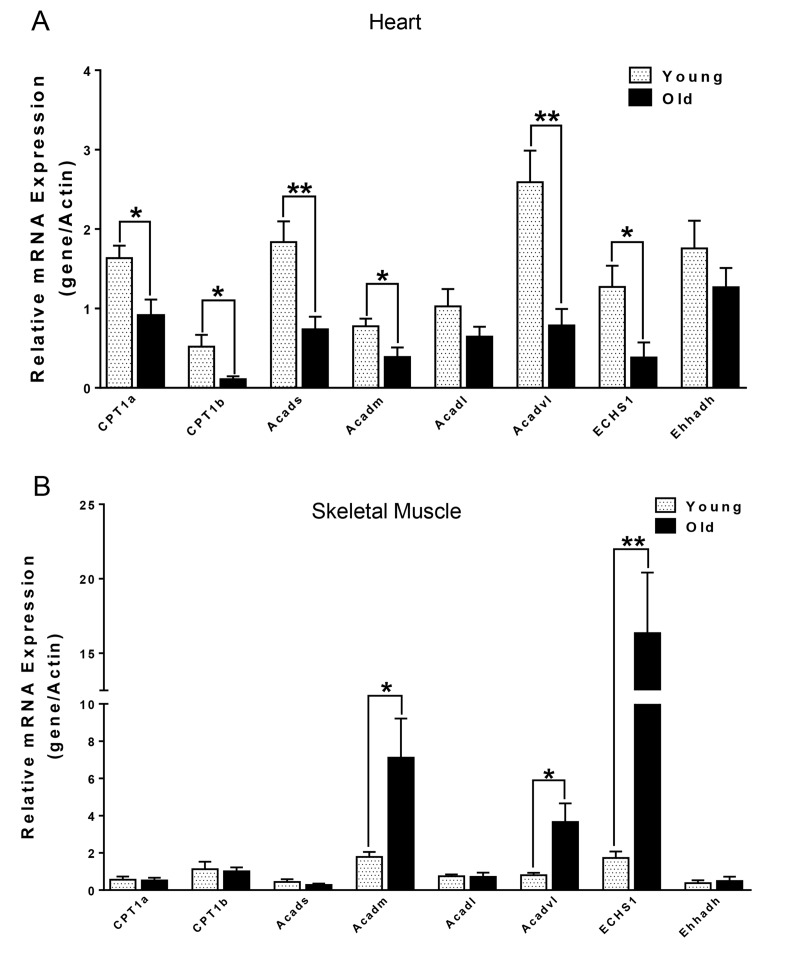
mRNA expression of CPT1α and β-oxidation enzymes in heart (A) and skeletal muscle (B) from young and old mice Values are means±SEM for 6 young and 5 old mice in each group. *P < 0.05.

**Figure 7:**
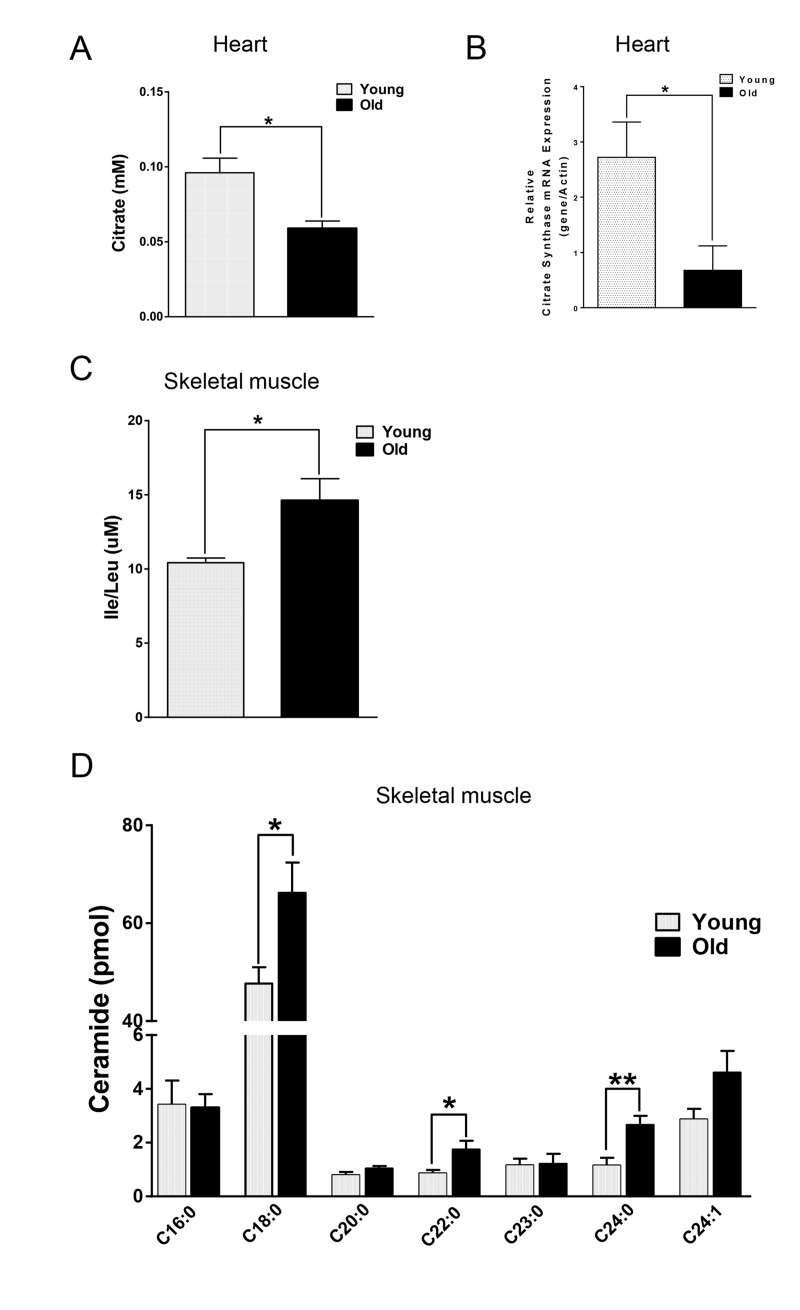
Differential changes of metabolites in skeletal and cardiac muscle from young and old mice (**A**) Decreased citrate concentration in the old heart. (**B**) Decreased mRNA expression of citrate synthase (CS) in old heart. (**C**) Increased branched chain amino acid (BCAA) in old skeletal muscle. (**D**) Increased ceramide in old skeletal muscle. Values are means±SEM for 6 young and 5 old mice in each group. *P < 0.05. **P < 0.01.

We next compared the metabolites in young and old skeletal and cardiac muscle by metabolomics profiling of acylcarnitines, TCA cycle intermediates, amino acids, and ceramides. In the heart tissue of aged mice, we observed decreased levels of some, but not all, species of short, medium, long, very long chain acylcarnitines (SCAC, MCAC, LCAC, and VLCAC, respectively; Fig. 5 A-D), indicating decreased fatty acid oxidation. Similarly, the mRNA expression of several enzymes involved in lipid oxidation: CPT1α and CPT1β, two isoforms of the enzyme responsible for the formation of acyl carnitines; acyl CoA dehydrogenase (Acads, Acadm, Acadl, Acadvl); and enoyl CoA hydratase (ECHS1, Ehhadh) all were down-regulated in the hearts from aged mice (Fig. 6A). Among the TCA intermediates, citrate was significantly less in the heart tissue from aged mice, and was associated with decreased mRNA expression of citrate synthase (Fig. 7A and B). In the muscles from aged mice, we observed less change in acylcarnitines than in the heart. However, there were still significant reduction in some acylcarnitine species (Supplementary Fig. S2). However, to our surprise, the mRNA expression of some of the β-oxidation enzymes including Acadm, Acadvl, Echs1 were significantly increased (Fig. 6B). Citrate also was increased, but not significantly, and citrate synthase mRNA expression was increased (Supplementary Fig. S3A and B). Interestingly, in the muscle from aged mice, we found increased levels of the branched chain amino acids, isoleucine and leucine (Fig. 7C). Ceramide levels also were increased, with C18:0 ceramide being the most abundant species and significantly increased in muscles from aged mice (Fig. 7D), whereas no changes were seen in the heart (Supplementary Fig. S3C and S3D). These particular changes have been shown to be associated with insulin resistance [24-27].

## DISCUSSION

Autophagy is required for maintaining skeletal muscle and heart function [28]. However, the reports about the effects of aging on autophagy in skeletal and cardiac muscles are inconsistent as the autophagosome marker, LC3B-II, has been shown to increase [1, 2], decrease [3], or remain unchanged [4, 5], and have led to conflicting views on the role of autophagy in these tissues during aging. The possible cause(s) of these discrepancies may be differences in species, age, and genetic background. To address these issues, we comprehensively compared the impact of aging on autophagy and cellular metabolism in murine skeletal and cardiac muscle, from the same strain of mice bred under identical conditions (Table 1 and Table 2).

**Table 1:** The differential changes of various autophagic markers in old skeletal and cardiac muscle

	Protein	Old skeletal muscle	Old cardiac muscle
Macroautophagy	p62	UP	UP
	ubiquitinated proteins	UP	UP
	LC3B-II /LC3B-I	DOWN	–
	ATG12-ATG5 conjugation	DOWN	–
	ATG3	DOWN	–
CMA	LAMP-2A	DOWN	UP
	Hsc70	DOWN	–
Mitochondrial quality control	Drp1	DOWN	–
	PINK1	DOWN	–
	PGC1 α	DOWN	–

**Table 2:** The differential changes of cellular metabolites in old skeletal and cardiac muscle

	Old skeletal muscle	Old cardiac muscle
Acylcarnitines	–	DOWN
Citrate	–	DOWN
Branched chain amino acid	UP	–
Ceramide	UP	–

Several lines of evidence in our studies point to decreased autophagy in both skeletal and heart muscles of aged mice. First, we found accumulation of p62 protein expression despite no change or decreased p62 mRNA expression in skeletal muscle and heart, respectively (Fig. 1A and B). p62 is a scaffold protein which binds to LC3B-II and marks ubiquitinated proteins for autophagosome-lysosomal degradation. Inhibition of autophagy results in accumulation of both p62 and ubiquitinated proteins in skeletal and heart muscles [3, 12, 20]. We observed that ubiquitinated protein levels were increased in both skeletal muscles and hearts from aged mice (Fig. 1E and F). However, the underlying mechanism for decreased autophagy appears to be different between these two tissues. In the muscle from aged mice, we observed decreased LC3B-II/LC3B-I ratio (Fig. 1A), indicating defective conjugation of phosphatidylethanolamine (PE) to LC3B. In contrast, there were no changes in LC3B-II/LC3B-I ratio in the heart. There are two ubiquitin-like systems that play very important roles in LC3B lipidation [19]. Among the relevant ATG proteins involved in these processes, we found decreased protein levels of ATG3 and ATG12-ATG5 conjugate (Fig. 2A and C), which have E2-like and E3-like enzyme activity for the LC3B lipidation process, respectively [29, 30]. Interestingly, the free form ATG5 also was increased and ATG12 remained unchanged (Fig. 2A), suggesting that there was a defect of the formation of the covalent bound between ATG12 and ATG5. ATG5 is one of the targets for deacetylation by SIRT1, a class III HDAC enzyme that is regulated in an NAD^+^-dependent manner [31]. It is possible that the decreased NAD^+^ concentration in muscle from aged mice [32] could explain the increased acetylation of ATG5 and decreased formation of ATG12-ATG5 conjugate. No changes in the protein and protein modification levels of these ATG proteins were found in the heart (Fig. 2B).

CMA is associated with age-related pathology such as the neuronal degeneration occurring in Alzheimer's and Parkinson's diseases [15, 16]. CMA was decreased in livers from aged mice due to the decreased level of CMA receptor, LAMP-2A [14]. Interestingly, transgenic expression of LAMP-2A restored hepatic CMA activity and function in aged animals [33]. However, the age-related changes in CMA in muscle and heart have not been characterized previously. In muscle from aged mice, we found decreased protein levels of Hsc70 and LAMP-2A (Fig. 3A), which could impair CMA capacity. Currently, the function of CMA in skeletal muscle is not well understood. Some studies suggested that the protein level of LAMP-2A in muscle increases in response to stress [34]; thus, it is possible that decreased expression of LAMP-2A leads to a functional decline in skeletal muscle function in aged mice. Of note, ryanodine receptor type 2, the major cardiac sarcoplasmic reticulum (SR) calcium-release channel is degraded by CMA [35]. Furthermore, starvation activates cardiac CMA [36]. In the aged heart, we found an increased level of LAMP-2A (Fig. 3B), suggesting that increased CMA may be compensating for the decreased general autophagy, or that defective CMA can lead to a compensatory increase of LAMP-2A level [16].

Mitochondrial quality control through mitochondrial turnover is comprised of the repair and/or removal of damaged mitochondria as well as the concomitant synthesis of new mitochondria. It is not clear whether there is a decline in mitochondrial quality control in muscle during aging. Interestingly, we found decreased protein levels of Drp1 and PINK1 in the muscle of aged mice (Fig. 4A). Drp1-mediated fission appears to be a crucial prerequisite for mitophagy, and the upregulation of Drp1 is associated with mitophagy during the early differentiation of muscle cells [37]. Thus, decreased Drp1 and PINK1 in the muscles from aged mice may lead to decreased mitochondrial fission and subsequent removal through autophagy. Substantial evidence also suggests that defective autophagy or mitophagy is associated with decreased PGC1α expression and mitochondrial biogenesis in a co-ordinate manner [23, 37]. In this connection, we found decreased PGC1α and COX IV mitochondrial protein levels in muscle from aged mice (Fig. 4B), suggesting that decreased mitochondrial biogenesis coincides with decreased autophagy/mitophagy. These effects led to impaired mitochondrial quality control in the muscle of aged mice.

Mitochondrial dysfunction can lead to changes in fuel utilization. Switches in cellular fuel utilization and metabolites are associated with metabolic disease conditions, including insulin resistance and type II diabetes. Muscle from aged mice has been shown to exhibit increased insulin resistance [38]. We also found several changes associated with insulin resistance by comparing the metabolites in the muscle tissues from young and aged mice. First, the branched-chain amino acid (BCAA) leucine and isoleucine were increased in muscle from aged mice (Fig. 7C). Of note, an association of BCAA and insulin resistance has been demonstrated in several cohort studies [24-26], and it has been suggested that BCAA themselves and/or their metabolites may be involved in development of insulin resistance [39, 40]. Second, intracellular ceramide level was increased in muscle from aged mice (Fig. 7D). Ceramide blocks insulin signaling and causes insulin resistance in cultured muscle cells [41]. Accumulation of ceramide previously was found in muscle samples obtained from obese and insulin resistant subjects [42], with the C18:0 ceramide as the most abundant species that was significantly increased in the diabetic muscle [43]. Interestingly, we found that the ceramide signature in the skeletal muscle of aged mice was similar to the one reported for muscle samples from diabetic patients [43] (Fig. 7D). Third, the mRNA levels of β-oxidation enzymes were increased (Fig. 6B), suggesting that skeletal muscle from aged mice may be trying to maintain fatty acid utilization, and thereby exhibited fuel inflexibility. Of note, muscle from elderly humans showed impaired de-phosphorylation of ACC and persistent fatty acid β-oxidation despite insulin stimulation [38]. Consistent with this fuel inflexibility, we found only a slight decrease in acylcarnitine levels in the skeletal muscle from aged mice (Supplementary Fig. S2). However, in contrast to muscle, heart tissue from aged mice showed a robust decrease in acylcarnitine levels (Fig. 5). The normal adult heart is highly dependent on fatty acid oxidation to produce energy, and decreased fatty acid oxidation is commonly observed in several heart failure models [44]. Here, we found decreases in the levels of numerous acylcarnitines including short, medium, long, and very long chain species, indicating the occurrence of deceased overall β-oxidation. In support of this finding, we also found decreased cardiac mRNA levels of all the acyl-CoA dehydrogenases responsible for different chain length fatty acids, and the acylCoA carrier proteins, CPT1 α and CPT1 β (Fig. 6A).

In summary, our study demonstrates that decreased autophagy occurs in both skeletal and cardiac muscles during aging, although the mechanism for the impairment in autophagy appears to be different between these tissues. Furthermore, there are decreased markers for CMA and mitochondrial quality control in the muscle whereas they are unchanged in the heart. The fuel preference and metabolism are differentially altered in these two tissues during aging, with skeletal muscle from aged mice showing a metabolomic signature suggestive of insulin resistance and fatty acid fatty acid fuel inflexibility whereas the heart exhibited decreased fatty acid β-oxidation. These differential effects in muscle and heart metabolism during aging suggest that different types of metabolic derangements may occur in muscle *vs*. heart, and thus may require different therapeutic approaches to optimize the function of these two tissues during aging and aging-associated diseases.

## METHODS

### Mice

Animal studies were conducted in accordance with the principles and procedures outlined in the National Institutes of Health Guide for the Care and Use of Laboratory Animals and were approved by the Institutional Animal Care and Use Committee (IACUC) of the South Texas Veterans Health Care System, San Antonio TX. Male C57BL/6J mice, born and reared in the San Antonio vivarium, were housed in hanging polycarbonate cages under a 12-hour light/12-hour dark cycle at 23°C with food and water available ad libitum.

Young (3.5 to 7 month old, with average of 5.1 month old) and aged (24 to 29 month old, with average of 27.6 month old) animals were sacrificed in CO2 chambers. Skeletal muscles and heart were collected in liquid nitrogen and subsequently used for protein and RNA isolation, and metabolomics analysis.

### Western blot analysis

Proteins were separated by SDS–PAGE under reducing conditions and transferred to polyvinylidene fluoride (PVDF) membranes. Membranes were blocked with 5% nonfat milk in phosphate-buffered saline with 0.1% tween 20 (PBST). The blots were incubated overnight at 4°C with primary antibodies. Immunoblot analysis was performed using an enhanced chemiluminescence procedure (GE Healthcare).

### RNA extraction and RT-PCR

RNA was isolated from muscle or heart using QIAzol (Qiagen), followed by clean-up on the Invitek Mini Kit (Invitek) following the manufacturer's protocol. RNA was quantified with a Nanodrop ND-1000 spectrophotometer. Total RNA (1 μg) was reverse-transcribed using iSCRIPT cDNA synthesis kit (Bio-rad) under condtions defined by the supplier. cDNA was quantified by real-time PCR on the Rotor-Gene Q System (Qiagen). PCR was performed using QuantiFast SYBR Green PCR Kit (Qiagen) according to manufacturer's instructions. Pre-designed KiCqStart^®^ Primers were purchased from Sigma-Aldrich.

### Metabolomics

Acylcarnitines, organic acids and amino-acids were extracted from 100 μL of tissue homogenate using methanol and then derivatized to form butyl esters using 3M HCl in butanol. Samples were then reconstituted in 80% aqueous methanol and 4 μL of this solution was injected into an Agilent SB-C8 column (12 × 50vmm with 1.8 um particle size) for analysis. Mobile phase used was 80% methanol and 20% water, and flow rate was maintained at 0.4 ml/min for 2 min. Isocratic flow of 0.6 ml/min of 30% acetonitrile and 70 % water with 0.1 % formic acid was maintained for 5.5 min.

### Statistics

Results were expressed as mean ± SEM. Statistical significance was calculated using GraphPad PRISM (version 6.0) software, taking p<0.05 as significant.

## SUPPLEMENTARY MATERIALS FIGURES


