# Supplementary material for: Changes in macroautophagy, chaperone-mediated autophagy, and mitochondrial metabolism in murine skeletal and cardiac muscle during aging
Source: Aging (Albany NY). 2017 Feb 26;9(2):583–96. doi: 10.18632/aging.101181 (PMC5361683; doi:10.18632/aging.101181)
Supplement: Supplementary file 1 [file aging-09-583-s001.pdf]

SUPPLEMENTARY MATERIALS

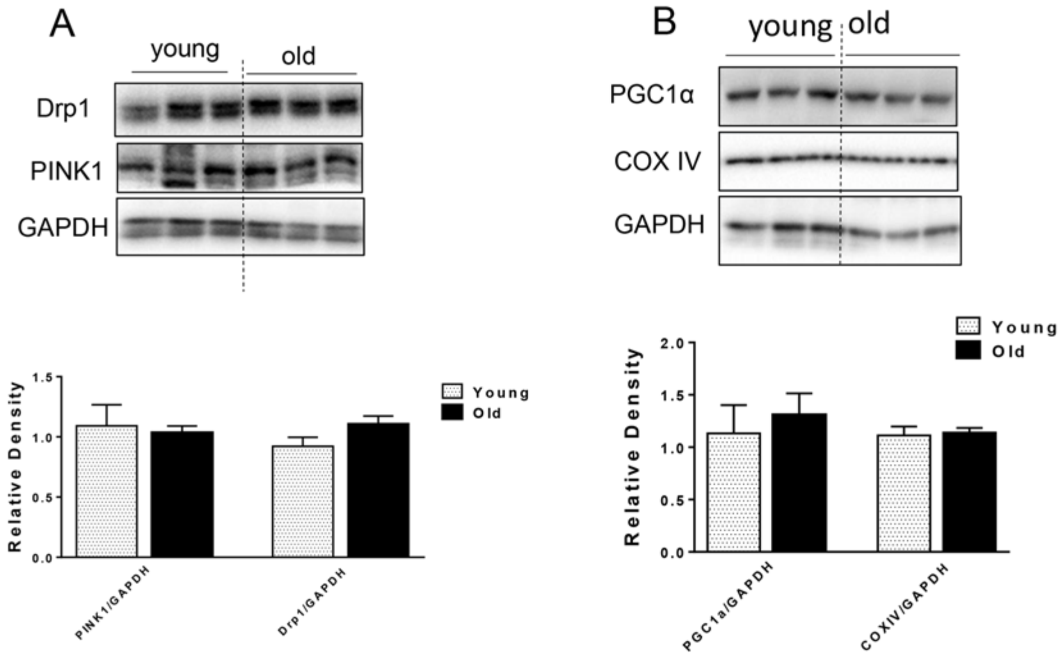

Supplementary Figure S1. The protein level of mitochondrial quality control markers in young and old heart. (**A**) Immunoblot and densitometric analysis of PINK1 and Drp1. (**B**) Immunoblot and densitometric analysis of PGC1 α and COXIV. Values are means±SEM for 6 young and 5 old mice in each group.

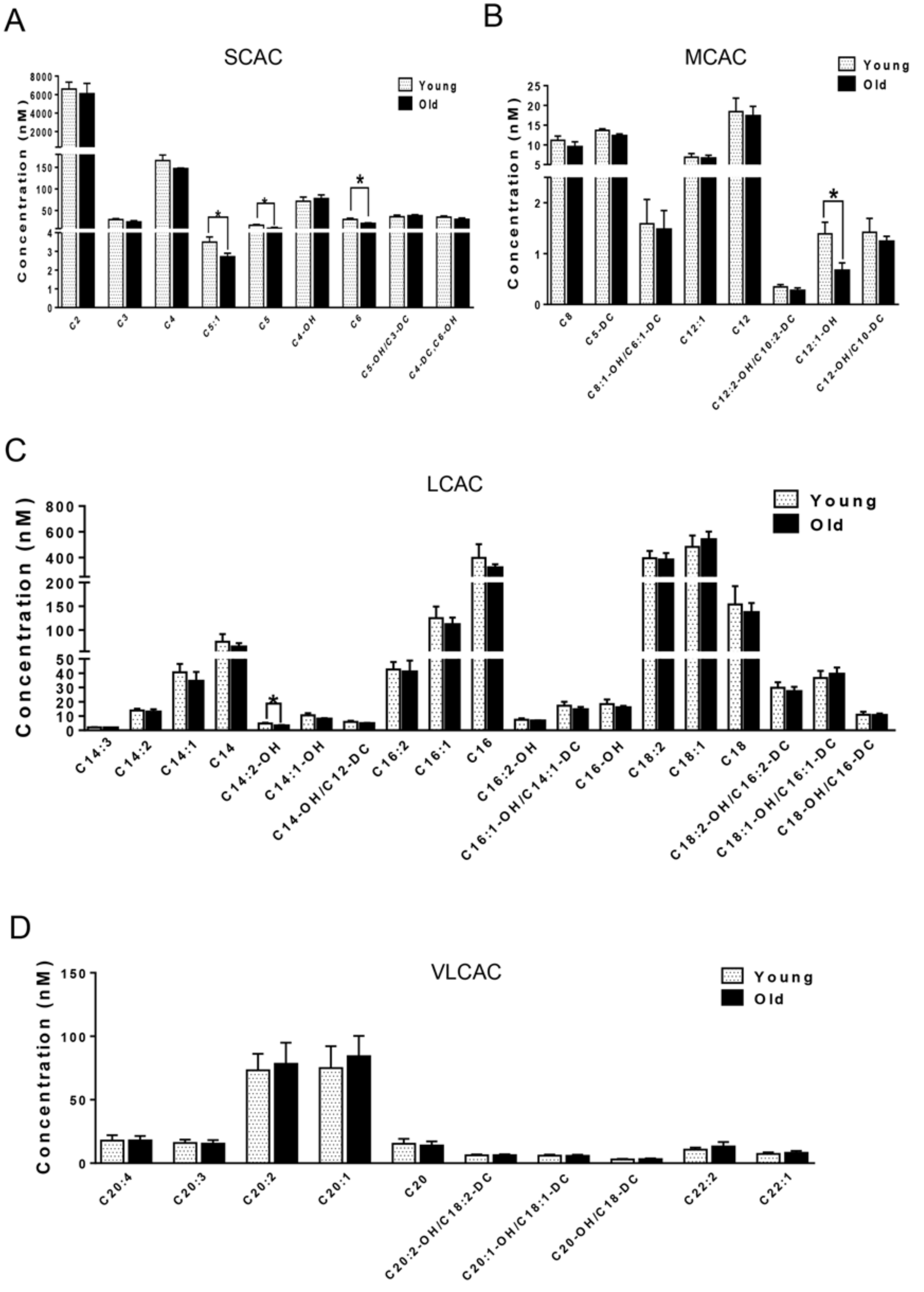

Supplementary Figure S2. Metabolomic analysis of acylcarnitines from skeketal muscle of young and old mice. (**A-D**) Metabolomic profiles of SCACs, MCACs, LCACs, VLCACs levels in cardiac muscle from young and old mice. Values are means±SEM for 6 young and 5 old mice in each group. *P < 0.05.

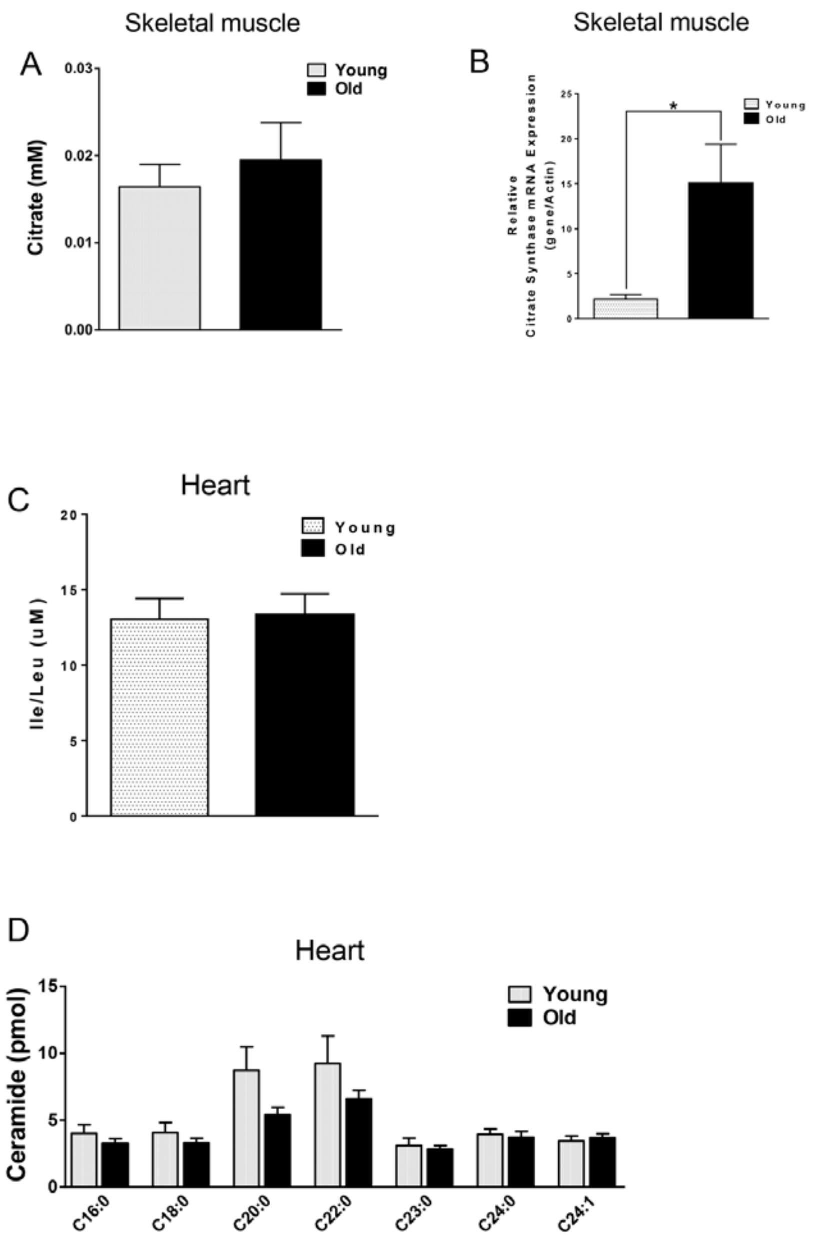

Supplementary Figure S3. Differential changes of metabolites in skeletal and cardiac muscle from young and old mice. (**A**) Citrate concentration in the young and old heart. (**B**) Increased mRNA expression of citrate synthase (CS) in old skeletal muscle. (**C**) Branched chain amino acid (BCAA) in the young and old heart. (**D**) Ceramide in young and old skeletal heart. Values are means±SEM for 6 young and 5 old mice in each group. *P < 0.05. **P < 0.01.
